# A mixed-methods evaluation of a novel targeted health messaging intervention to promote COVID-19 protective behaviours and vaccination among Black and South Asian communities living in the UK (the COBHAM study)

**DOI:** 10.1016/j.clinme.2025.100285

**Published:** 2025-01-23

**Authors:** Katie Sutton, Jo Armes, Lindsay Forbes, Amran Mohamed, Shuja Shafi, Reham Mustafa, Sunayana Shah, Andrew Hayward, Tasneem Pirani, Tushna Vandrevala, Jane Hendy, Osman Dar, Miqdad Asaria, Alimuddin Zumla, Aftab Ala

**Affiliations:** a School of Health Sciences, Faculty of Health and Medical Sciences, University of Surrey, Guildford, UK; b Centre for Health Service Studies, University of Kent, Canterbury, UK; c Access and Medicine, Royal Surrey NHS FT, Guildford, UK; d Mass Gatherings and Global Health Network, London, UK; e Independent Pharmaceutical Consultant, London, UK; f Institute of Epidemiology and Health, University College London, London, UK; g Critical Care, Institute of Liver Studies Kings College Hospital, London, UK; h Faculty of Health, Science, Social Care and Education, Kingston University of London, London, UK; i Kingston Business School, Kingston University of London, London, UK; j UKHSA and Chatham House, London, UK; k Department of Health Policy, London School of Economics and Political Science, London, UK; l REAL Centre, The Health Foundation, London, UK; m Research and Documentation Committee, Muslim Council of Britain, London, UK; n Department of Infection, Division of Infection and Immunity, University College London, London, UK; o Biomedical Research Centre, UCL Hospitals NHS Foundation Trust, London, UK; p Institute of Liver Studies, Kings College Hospital NHS FT, London, UK

**Keywords:** COVID-19, Protective health behaviours, Vaccination, Targeted health messaging, Ethnic minority

## Abstract

**Aim:**

To evaluate an intervention (a film and electronic leaflet) disseminated via text message by general practices to promote COVID-19 preventative behaviours in Black and South Asian communities.

**Methods:**

We carried out a before-and-after questionnaire study of attitudes to and implementation of COVID-19 preventative behaviours, and qualitative interviews about the intervention, with people registered with 26 general practices in England who identified as Black or South Asian.

**Results:**

In the 108 people who completed both questionnaires, we found no significant change in attitudes to and implementation of COVID-19 preventative behaviours, although power was too low to detect significant effects. A key qualitative finding was that participants felt they did not ‘belong’ to the group targeted by the intervention.

**Conclusion:**

Interventions targeting ethnic minorities in the UK need to acknowledge the heterogeneity of experience and circumstances of the target group so that people feel that the intervention is relevant to them.

## Highlights

• Some participants did not feel that the intervention was targeted at them, highlighting the heterogeneity of the ethnic minority experience.
• Future health promotion interventions need to acknowledge the diversity of minority ethnic communities.
• The content of health promoting interventions needs to address different ways of changing behaviour that might be effective in different subgroups.
• Health promotion interventions must avoid othering to build trust.
• Our evidence can inform the design of future interventions to promote preventative behaviours in relation to communicable disease control in people from ethnic minorities living in the UK.

## Introduction

During the COVID-19 pandemic, Black and South Asian people in the UK experienced a significantly higher burden of morbidity and mortality than White counterparts.^1^^,^ ^2^ Vaccine hesitancy and barriers to vaccine uptake were also more common among some minority ethnic communities than White communities.^3^, ^4^, ^5^ This prompted the need to rapidly develop culturally appropriate materials targeted at people from minority ethnicities in the UK to promote COVID-19 preventative measures.^6^

In collaboration with partners from Black faith and community groups, the Muslim Council of Britain, Hindu, Buddhist and Christian groups among others, we created an intervention comprising two short films and an e-leaflet to provide information and advice on COVID-19 preventative behaviours for Black and Asian populations, which can be accessed here: https://www.royalsurrey.nhs.uk/ethnic-community-covid-19-study.

We report our findings from our mixed-methods evaluation of this intervention. The evaluation protocol has been published elsewhere by our group (the COBHAM study).^7^

## Aims

We aimed to answer the following research questions:

1. Does the effect of the intervention increase confidence in intentions to carry out COVID-19 preventative behaviours among Black and South Asian people?
  • Delays in obtaining governance approvals meant that, by the time we were able to collect data, the overwhelming priority among COVID-19 preventative behaviours was vaccination, so we focused our analysis on that issue.
2. How do Black and South Asian people understand and interpret COVID-19 health messages in the intervention?

## Methods

We carried out:

• questionnaires assessing intentions and confidence to carry out COVID-19 preventative behaviours, before and after the interventions
• qualitative interviews exploring the views of Black and South Asian people on the interventions’ messages and on carrying out COVID-19 preventative behaviours.

We originally planned to collect data from health professionals and to carry out focus groups in addition, but delays in governance approvals meant that this was not feasible.

As we aimed to roll out the intervention as quickly as possible during the pandemic, we did not have time to generate sufficient data to inform what the size of effect might be.

Our sample size was determined by the number of practices and the number of participants agreeing to take part. At the time of designing the study, we knew that at least five to seven large practices (with a total population approximately up to 40,000 registered patients) had agreed to take part. These practices are all known to have a relatively high proportion of people from the Black or Asian communities. Therefore, we felt that it was reasonable to aim for up to 600 participants who had not yet been vaccinated to provide data on both questionnaires.

We recruited general practices through NIHR Local Clinical Research Networks, who, between 3 September 2021 and 31 January 2022, invited all registered eligible patients (aged 18+, recorded as being of Black or South Asian ethnicity) to take part using secure one-way text messaging. The text message provided a hyperlink for further information, a consent form and an electronic questionnaire (Qualtrics Software Version October 2021, © Qualtrics 2020, Provo, UT, USA. https://www.qualtrics.com). At the end of the questionnaire, the participant was invited to click on a link to see the films and e-leaflets and provide a mobile phone number to allow further contact. The research team sent a second electronic questionnaire to those who provided a mobile phone number, a week after receiving the first. This repeated questions about intentions and confidence to carry out COVID-19 preventative behaviours and also asked for opinions on the intervention, including closed and free text questions.^7^

We linked responses to the questionnaires before and after the intervention, using the mobile phone number as the unique identifier. We identified people as ‘vaccine hesitant’ if they reported no vaccination *and* either did not intend to take up vaccination or were indecisive about doing so.^8^ We identified people as ‘not confident’ in vaccination if they answered ‘not at all confident’ or ‘not very confident’ to the question ‘How confident are you that the COVID-19 vaccination programme will benefit people from Black and Asian communities in the UK?’. We calculated the proportions who changed their vaccine hesitancy or confidence in vaccination before and after the intervention and tested the differences using McNemar’s test. Details about questionnaire development and sample size are provided in our protocol publication.^7^ The content of the intervention was based on rapidly evolving national guidance from Public Health England and the Office of the Chief Medical Officer rather than a specific theoretical framework. This was felt to be the most appropriate approach to design, given the ever-changing nature of public health messaging during the pandemic.

We recruited participants for the qualitative study by asking post-intervention questionnaire respondents to participate. Those who agreed to be approached were provided with further information. After informed consent, we carried out interviews via Microsoft Teams or telephone (all audiorecorded) using a topic guide (see supplementary material).^7^ Participants’ time was recompensed with a £25 voucher. Interviews were conducted by KS, who has extensive experience in qualitative interviewing techniques. We maximised variation in our sample by purposively recruiting people from a variety of ethnicities, genders, age groups and vaccine intentions to ensure information power.^9^ Interview transcriptions were downloaded from Microsoft Teams, and data were analysed to identify themes using Framework Analysis,^10^ using NVivo software (v1.6). Two members of the research team (KS and JA) coded the transcripts independently and then compared coding to achieve congruence.

## Results

We asked 26 primary care practices to participate through the NIHR Local Clinical Research Networks across England. The practices sent text messages about the intervention and evaluation to 42,515 eligible people registered at their practices. Fourteen practices were in London, seven in northern England, two in southern England, two in the Midlands and one in eastern England.

Table 1 provides details of the demographics of participants. 651 people responded to the first questionnaire (pre-intervention). Most respondents (n=504;77%) were aged under 60; 354 (54%) were women; 332 (51%) were Black, 249 (38%) South Asian and 70 (11%) were of mixed, other or unknown ethnic group; 218 (33%) were British-born. 527 (81%) participants provided a valid phone number and were sent the post-intervention questionnaire; 130 (25%) responded to this. 108/130 (20%) provided a valid mobile number, allowing linkage with pre-intervention questionnaire data.

**Table 1:** Demographics of participants.

		*Pre-intervention questionnaire*		*Interviews*
		n	(%)	n
Age group	18–39	249	(38.3)	5
	40–59	255	(39.2)	11
	60+	105	(16.1)	4
	Missing	42	(6.5)	
				
Sex	Female	354	(54.4)	11
	Male	251	(38.6)	9
	Other/prefer not to say/missing	46	(7.1)	
				
Ethnic group	Black	332	(51.0)	10
	South Asian	249	(38.3)	10
	Other/missing	70	**(10.8)**	
				
Place of birth	UK	218	(33.5)	
	Africa	192	(29.5)	
	South Asia	124	(19.0)	
	Other or missing	117	(17.9)	
				
Religion	Christian	297	(45.6)	
	Muslim	129	(19.8)	
	Hindu	84	(12.9)	
	Sikh	14	(2.2)	
	No religion	60	(9.2)	
	Other or missing	14	(2.2)	

20 qualitative interviews were conducted. Three people we contacted refused to participate (one had a family bereavement and two did not give a reason for not wanting to be involved) and two agreed but did not attend the interview. Interviews lasted between 12–40 min. The qualitative data table is provided as supplementary information.

**Fig. 1:**
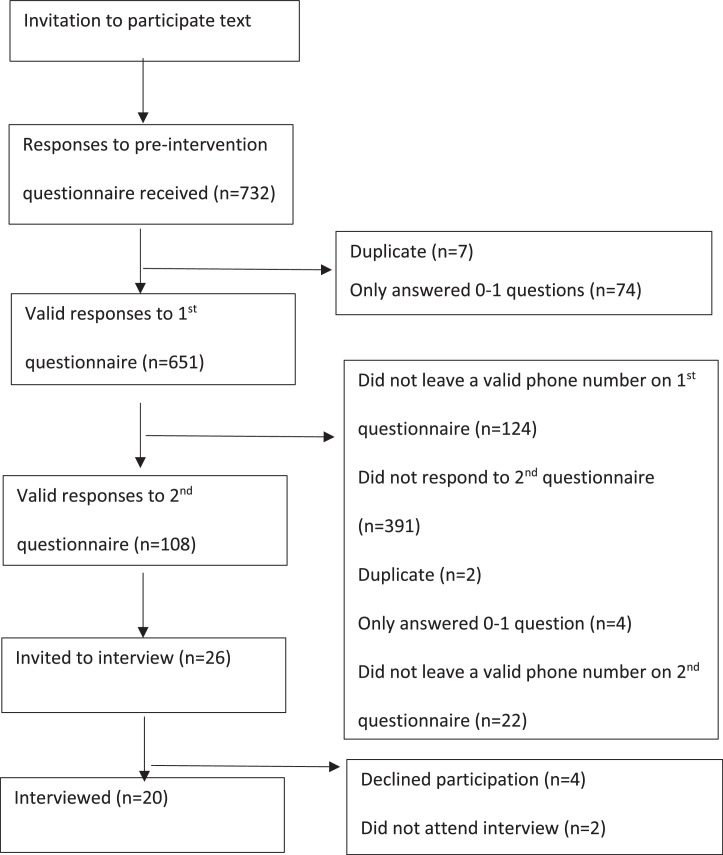
Participant flow chart.

Fig. 1 *shows a flowchart for participation from 26 practices, which sent text messages to 42,515 registered patients. We received valid responses to the pre-intervention questionnaire from 651 people, from which a total of 20 qualitative interviews were conducted with participants recruited via the questionnaire regarding their impressions of the film and its potential impact on their behaviour*

### Vaccine hesitancy and confidence in the vaccination programme before receiving the intervention

Most questionnaire respondents (n=588; 90%) said that they had been vaccinated or planned to be vaccinated. 60 (9%) were vaccine hesitant: 28 (47%) said that they were worried it would make them ill, 27 (45%) said that they were not sure it was safe, 15 (25%) said that they believed they were already immune, seven (12%) said that they did not think it would work, and six (10%) said that they did not believe in vaccines. One reported that it was too difficult to get vaccinated.

Table 2 shows the prevalence of vaccine hesitancy in the pre-intervention questionnaire. Black people were much more likely to be vaccine hesitant than South Asian people (42/332 vs 8/249; 13% vs 2%; *p*<0.01). Within the South Asian group, there were no statistically significant differences in vaccine hesitancy by age, sex or place of birth (although the numbers were small). Within the Black group, vaccine hesitancy was most common in the 18–39 age group (21%) and those born in the UK (20%).

**Table 2:** Prevalence of vaccine hesitancy among participants in the first questionnaire (pre-intervention).

		Black (n=331)			South Asian (n=247)		
		n	(%)		n	(%)	
Age group	18–39	22	(21.2)		4	(3.1)	
	40–59	19	(11.7)		4	(5.1)	
	60+	1	(1.2)		0	(0.0)	
*p*-value for age group				*p<0.01*			*p=0.37*
Sex	Male	15	(12.7)		4	(3.3)	
	Female	27	(12.7)		3	(2.4)	
*p*-value for sex				*p=0.97*			*p=0.72*
Place of birth	UK	24	(20.2)		3	(3.6)	
	Not UK	17	(8.7)		5	(3.4)	
*p*-value for place of birth				*p<0.01*			*p=1.0*

### Change in vaccine hesitancy and confidence in the vaccination programme after the intervention

In the 108 people with questionnaire data both pre- and post-intervention, we found no statistically significant change in vaccine hesitancy (9/108 vs. 9/108) or confidence in the vaccination programme.

13 interview participants said that watching the film did not have any impact on their behaviour. For 11 of them, this was because they felt that they were already undertaking the preventative behaviours suggested. Two said that the intervention did not offer any new information that might change their perspective, and one said that they did not think it would have an impact on people who were resolutely anti-vaccination. Two women mentioned that it would not change their minds, as it did not address their concerns (fertility and needle phobia). Two participants stated that even though they were already doing as advised in the video, they felt that it was beneficial to watch as it reassured them that they were acting appropriately.

### How do Black and South Asian people interpret and understand the intervention?

The following theme descriptions integrate quantitative and the qualitative findings. Relevant quotations for each theme, as well as the qualitative coding tree, are presented in tables 3 and 4 (supplementary material).

#### Overall perceptions of the intervention

79 (61%) of the 108 questionnaire respondents for whom pre- and post-intervention linked data were available gave an overall opinion of the intervention as very good or fairly good; 88 (71%) said that they remembered very well or quite well what the film was about; 63 (49%) said that they were very or fairly likely to recommend the film to friends and family, and 29 (22%) said that they had shown the film to family and friends (see table 5-supplementary material). In free text responses, there were many positive comments, with an overall sense that people felt that it was clear and understandable.

During the qualitative interviews, ten participants felt that the video was *‘good’*, informative, with accessible and clear information. It offered a good summary and a useful reminder of what people needed to do to protect themselves, and that it was *‘encouraging rather than scary’*. Others noted that it was *‘short and snappy’, ‘well-presented’, ‘professional and informative’*. Six participants spoke positively of the representation of people from ethnic minority backgrounds in the film.

Three people commented that the video was patronising or condescending, and that it was about forcing people to have the vaccine. Five said that it did not offer any new information, and therefore did not address their concerns. A further three thought that it did not have enough impact to change behaviours.

In the interviews, we asked whether participants perceived any aspects of the video to be offensive, to which 18 of the 20 reported that they did not. However, one said that they found it insulting, and another said that the video gave the impression that if they did not participate in the COVID vaccine programme they would be perceived an ‘uncaring and nasty’ person.

#### The ethnicity-specific nature of the intervention

There were conflicting opinions during the interviews on the ethnicity-targeted approach of the film. Six participants commented positively on this targeted approach, with some saying that this approach was necessary because of lower levels of vaccine uptake, and so sending the message that people from their community had been vaccinated might impact positively on the choices of those who watched. There was also a suggestion that such representation may lead to feeling pride in the member of their community taking part in the film.

Conversely, both the free text comments from the questionnaire and discussions during the interviews suggested some anger and disappointment about the fact that the video was targeted at specific ethnic groups. They questioned why a different approach was warranted for certain ethnicities and would have preferred to see one film that included all ethnic groups. The words ‘*patronising*’ and ‘*condescending*’ were used by participants in both the questionnaires and the interviews to describe their response to the intervention, and it was suggested that having one film that included a broad range of ethnicities rather than a targeted approach would be less stigmatising.

During the qualitative interviews, one participant spoke extensively and passionately about how they found the film condescending and felt patronised by what they perceived to be a message that suggested people from their community specifically needed telling to wash their hands. This objection to an ethnicity-specific approach was echoed by another participant, who suggested that it highlighted differences between groups in a negative way. Similarly, one person stated that they would prefer the leaflet to include a variety of ethnicities rather than being targeted, and the fact that it was targeted was a disappointing reflection of society.

#### Suggestions for improvement to the film

During the interviews, seven participants commented on the need for more facts about COVID-19, including those that address misinformation and people’s fears in a more hard-hitting fashion. Two interviewees suggested including evidence about other illnesses and vaccinations as a means of offering reassurance, and responses from both the questionnaires and the interviews suggested that they would like to hear more from experts such as scientists and medical professionals. Three interviewees thought that the inclusion of religious leaders would be beneficial for some, as they were trusted figures in society.

Two interview participants suggested the video would be best shared in mosques and on Asian TV channels, and some people highlighted the need to present the video in a variety of languages. Seven participants thought that the video should include more younger people and should also include more famous women, as well as figures from the worlds of sport and music.

#### Who would benefit from seeing the film

During qualitative interviews, generally, younger participants thought that older people would benefit most from watching the video and vice versa. Three participants thought the older generation would respond most positively to seeing their communities represented. Conversely, three others thought that younger people would benefit most, as they were most likely to be unvaccinated and might not be taking COVID-19 seriously enough. Some of the younger participants suggested that they would not see the need for an ethnic community-specific film and would rather see a diverse mix in a video aimed at everyone. Three reported that it was those who were less informed, perhaps without access to television, news and the internet, who would find the video useful. Three felt that the video would have the most impact on people who were neutral or undecided about having a vaccination. Four interviewees commented that it was those who are the most sceptical about vaccination who would benefit the most, but also that they were also the least likely to watch it.

While some interview participants spoke positively about the film, they did not perceive that it was aimed at them, but rather at other specific communities within their ethnic group. One participant said that they did not feel a part of the group of people that the film was directed at. Another also mentioned that there were different Asian communities, and they did not feel like they were a part of the community that needed to see the video.

## Discussion

We found no evidence that the intervention changed hesitancy or confidence in relation to COVID-19 vaccination. Among Black people, we found marked differences in vaccine hesitancy by age group and country of birth – the most likely to be vaccine-hesitant groups were young Black people born in the UK. We also found that not all participants felt that they ‘belonged’ to the group targeted by the intervention, because of the heterogeneity in people’s experience, depending on their age, culture and background.

In this study, age and generational differences were seen to be as important a factor as ethnicity when it came to developing a targeted intervention. This finding was similar to that of Benham *et al*,^11^ who also found that while most participants in their study thought that young people were least likely to follow social distancing rules, younger people believed that the older generation were less likely to adhere to preventative measures.

There was evidence in our study that the targeting of specific communities heightened feelings of hostility and stigmatisation among some participants. There was a suggestion that it felt as if groups we were targeting were to a certain extent being blamed for the increased prevalence of the disease, and that directing messages about preventative behaviours at them implied that they were at fault, or ‘a problem’, and risked increasing feelings of segregation or othering.^5^^,^ ^10^ The risk of stigmatisation resulting from targeted messaging has been identified elsewhere, particularly when an inaccurate perception of cultural norms contributing to a public health risk develops.^12^ This approach may even serve to heighten inequality and have a negative effect on adherence to preventative measures resulting from feelings of anger and frustration.^13^

Involving healthcare workers and community leaders from minority ethnic backgrounds was also identified in our study (and has been reported elsewhere) as a potential approach to address misinformation and enhance trust and acceptability of a public health message.^14^, ^15^, ^16^, ^17^ We recognise that more studies are needed to understand this further.

### Strengths and limitations

The intervention was disseminated to a very large number of people very quickly through using primary care text messaging systems. However, we cannot measure how many of these viewed the intervention. Although our analysis did not indicate an effect of the intervention on COVID-19 preventative behaviours, vaccination confidence and hesitancy, we cannot conclude that the intervention had no effect because the small survey sample size meant very low power. While our response rate of only 20% is significant for our results, this is usual for surveys of healthy people and is similar to the response rate achieved by the GP patient survey.^18^

There are specific limitations to our study relating to the challenges of conducting research during the height of the COVID-19 pandemic. By the time that we were able to collect data, vaccine uptake and adherence to preventative behaviours had significantly increased. The urgency of the public health crisis also led us to take pragmatic methodological steps to expedite evaluating our intervention. These included the use of an unvalidated questionnaire, resources being available in English language only and a lack of information for sample size calculations.

Our study may have been subject to a degree of responder bias,^19^ as we speculated that participants who agreed to a qualitative interview were more motivated to be involved in the pandemic response or have strong opinions for or against vaccination, with a likely under-representation of people ‘on the fence’. It is also important to note that the researcher conducting the interviews was a White British woman. While we cannot ascertain the impact that this may have had on our qualitative findings, co-facilitation with people from Black and South Asian communities may have been valuable had time allowed.

## Conclusion

The COBHAM study involved the development and evaluation of an intervention to promote preventative behaviours among Black and Asian people during the COVID-19 pandemic. Our study underwent a dynamic evolution due to the rapidly shifting public response to the pandemic and an increased uptake in preventative behaviours over time. In line with these changes, we did not find that the intervention had an impact on vaccine hesitancy or confidence in the COVID-19 vaccination programme, although power was low. Our study did reveal interesting findings about the perceptions of Black and South Asian people regarding targeted interventions, suggesting that such approaches to public health messaging require an awareness of the potential risk of consolidating feelings of othering and stigmatisation. Careful consideration of issues such as institutional mistrust and intersectionality are also fundamental to the development of public health messages that will be both beneficial and well received by people from different ethnic communities.

## Summary box

**Table:** 

***What is known?***	During the COVID-19 pandemic, Black and South Asian people in the UK experienced a significantly higher burden of morbidity and mortality than their White counterparts. Vaccine hesitancy was also more common among minority ethnic communities than White communities.
***What is the question?***	An evaluation of a novel targeted health messaging intervention to promote COVID-19 preventative behaviours and vaccination among Black and South Asian communities living in the UK
***What was found?***	We found: • no significant impact of the intervention on vaccine hesitancy, or confidence in the COVID-19 vaccination programme, although statistical power was low • the intervention was perceived by some participants not to be targeted at themselves, highlighting the heterogeneity of the ethnic minority experience.
***What is the implication for practice now?***	Our evidence can inform the design of future interventions to promote preventative behaviours in relation to communicable disease control in people from ethnic minorities living in the UK: • the need to acknowledge the heterogeneity of minority ethnic communities in terms of age and ethnic subgroup, for example. The content of health promoting interventions needs to address different ways of changing behaviour that might be effective in different subgroups • the need to avoid othering to build trust.

## Ethics approval and consent to participate

The study was approved by the London – Brighton and Sussex Research Ethics Committee (REC reference number 21/LO/0452) in June 2021. Research and Development Approval and sponsorship for the study was obtained in August 2021 from Royal Surrey NHS Foundation Trust. The consent process (approved by the ethics committee) for the questionnaires and interviews was as follows:

Questionnaires: Participants were sent a text with a link to online consent.

Interviews: Was a follow-on from the questionnaires where participants consented online to being contacted. Then during the interview, video-recorded informed consent was obtained.

## Funding

This research was funded by the National Institute for Health and Care Research (DHSC/UKRI/MRC) COVID-19 Rapid Response Initiative, Developing and delivering targeted SARS-CoV-2(COVID-19) health interventions to Black, Asian and minority ethnic (BAME) communities living in the UK (Grant Award Number COV0143). The views expressed in this publication are those of the author(s) and not necessarily those of the National Institute for Health and Care Research or the Department of Health and Social Care.

Jo Armes and Lindsay Forbes receive funding from the NIHR Applied Research Collaboration Kent, Surrey, Sussex.

Aftab Ala receives funding from the NIHR and Innovate UK.

## Data availability statement

The data that support the findings of this study are available from the corresponding author upon reasonable request.

## CRediT authorship contribution statement

**Katie Sutton:** Writing – review & editing, Writing – original draft, Visualization, Validation, Software, Resources, Project administration, Formal analysis, Data curation. **Jo Armes:** Writing – review & editing, Validation, Supervision, Methodology, Funding acquisition, Formal analysis. **Lindsay Forbes:** Writing – review & editing, Validation, Methodology, Investigation, Formal analysis. **Amran Mohamed:** Writing – review & editing, Validation, Software, Resources, Project administration, Methodology, Investigation, Data curation. **Shuja Shafi:** Writing – review & editing, Methodology, Investigation, Funding acquisition, Conceptualization. **Reham Mustafa:** Writing – review & editing, Software, Resources, Project administration, Investigation, Data curation. **Sunayana Shah:** Writing – review & editing, Funding acquisition. **Andrew Hayward:** Writing – review & editing, Investigation, Funding acquisition. **Tasneem Pirani:** Writing – review & editing, Investigation. **Tushna Vandrevala:** Writing – review & editing, Funding acquisition. **Jane Hendy:** Writing – review & editing, Funding acquisition. **Osman Dar:** Writing – review & editing, Funding acquisition. **Miqdad Asaria:** Writing – review & editing, Funding acquisition. **Alimuddin Zumla:** Writing – review & editing, Funding acquisition, Conceptualization. **Aftab Ala:** Writing – review & editing, Visualization, Validation, Supervision, Software, Resources, Project administration, Methodology, Investigation, Funding acquisition, Formal analysis, Data curation, Conceptualization.

## Declaration of competing interest

The authors declare the following financial interests/personal relationships which may be considered as potential competing interests: Aftab Ala reports financial support was provided by Royal Surrey NHS Foundation Trust. Aftab Ala is an Associate Editor of Clinical Medicine. If there are other authors, they declare that they have no known competing financial interests or personal relationships that could have appeared to influence the work reported in this paper.

## Appendix Supplementary materials

Image, application 1
