# Supplementary material for: A mixed-methods evaluation of a novel targeted health messaging intervention to promote COVID-19 protective behaviours and vaccination among Black and South Asian communities living in the UK (the COBHAM study)
Source: Clin Med (Lond). 2025 Jan 23;25(2):100285. doi: 10.1016/j.clinme.2025.100285 (PMC11876826; doi:10.1016/j.clinme.2025.100285)
Supplement: Supplementary file 1 [file mmc1.docx]

**COBHAM paper-supplementary tables and figures**

Table 3: Qualitative data

| Theme | Qualitative interview data | Questionnaire free-text data |
| --- | --- | --- |
|  |  |  |
| Perceptions of the film | *So good to see people of different races, different colours in the video, so it stands out, I think. Yeah, that's good actually. (P20)* | *I think it was a good idea for using black and asian people in the film which makes it strong to get the message through.*  *I think the film was clear in explaining why the need to encourage BAME communities to take the jab; keep themselves and society in general safe.*  *The casts for both films are very representative of the two groups of communities and this makes the viewer feel they are part of the film* |
| The ethnicity-specific nature of the film | *So good to see people of different races, different colours in the video, so it stands out, I think. Yeah, that's good actually. (P13)*  *I think you have to. You have to break it down because each community has different needs and different ways of looking at things. You have to let people from different communities see that people like them are agreeing with getting the vaccine done. (P20)*  *It also represents my communities, someone from my community has been made an ambassador to promote certain things. It makes one feel proud of it. (P20)*  *Well, as with a lot of media that's aimed at African and Asian communities. It's quite condescending, really…Washing hands is not you know, to be told you need to start washing your hands. It's like we've never washed our hands before……*  *All you're doing is, you're insulting us. You're insulting our intelligence by trying to trail out different stereotypical views. You know images of black people.*  *Diversity isn't about making sure that you have one of each type. Diversity is about making sure that you demonstrate integration.*  *You're not talking to me as an adult, as a black man in this time aware of things, you're talking to me as somebody who just come off the boat…Is this somebody who is fresh come off the boat from Africa or Jamaica? Who doesn't know anything about the UK...*  *When you converse with us, converse with us as people who know what's going on and who need to be informed in the same way. As if you would also be conversing with other native white English people, yes, because that's the sense a lot of people get. The sense that we get a different narrative. (P1)*  *I feel like I don't need to be focused [on] separately. I'm fully integrated. You don't need to be putting actors of Asian community or black community. It should be open to everyone. There shouldn't be any form of separation. It's one for all. (P9)*  *I just wanted to see if there's a sheet that's being disseminated to the masses, that the faces correspond to community instead of just being ‘OK these are for white faces. These are for brown faces.’ Can't we just have one sheet? …It is sad that it's required. It is sad that we just don't see people. (P5)* | *I thought the message was good but I kind of felt like your entire survey + video + leaflet has suggested that it is Asian people's fault that they are suffering worse from covid (as if we don't wear face masks as much). When really there are serious racial discriminations at play in British society which is making it more likely for Asians to catch covid and then not receive proper healthcare to cope with it. Maybe some people of Asian descent have jobs where they cannot work from home or they need to travel on public transport more etc.?*  *Really fed up with people calling coloured people as minorities and categorising based on colour and target market them.*  *As we all follow the same rules, don’t see the need of a separate film for ethnic minorities Black and South Asian. It is the same rule for Caucasian too.*  *I found it quite condescending.*  *Patronising, embarrassing and will turn off your target audience. Fact.*  *Patronising. Assumes the target audience is not educated.* |
| Suggestions for improvement to the film | *perhaps someone from the sporting world on there that was fully fit, didn't have the vaccine, caught COVID, messed them up. And they wished they took the vaccine. So I think explain that you could be fully fit, [but] you won't necessarily fight it like you think. (P5)*  *Pictures of people in hospitals. I'm looking for facts. Not acting. Real people, real, you know, real suffering, not acting. (P2)*  *Maybe some real people could be in it, like who's really had COVID. (P14)*  *perhaps real life stories in there…there's others who obviously suffered terribly because they haven't [had the vaccination] and I just think we've got to somehow capture that story to convince people you know. (P11)*  *If you were actually to show people who were in a hospital bed, who you know gave real testimonies to say you know, uh, boy I wish I had, you know Uh, taken up some advice or I should have done this sooner then I think that hits home more. (P1)*  *There's nothing there that's actually dealing with my biggest fear, whatever that might be. (P11)*  *It's got to be a bit harder than a cuddle. (P11)*  *I'm thinking maybe that's a way to approach the video where it literally is like a, you know a box pops up… A series of questions..you know I'm worried about this 'cause you're gonna put 5G in me or whatever it is…Then it's actually dealing with the fears in the video. (P16)*  *But there was no evidence in there was there, it was just all wash your hands, get the vaccine. That is what everybody is asking for. We need more information. (P16)*  *Having some references to say hey this kind of misinformation is going on. Please don't believe in this. Present facts...say Don't believe those things. (P20)*  *The older people would understand if they could relate it to something that's happened when they were back where they come from. I'm from India, so if it's from India, for example they will have gone through experiences where they would have had infections going on in their life, like polio vaccines and things like that. You know in their life they’ve come across a lot of diseases, but not COVID. They won't know what COVID means. (P7)*  *give examples of other vaccinations or even other medical trials something like that So people can put things in perspective. (P5)*  *I think the lead in the film should be a medical professional in terms of saying. You know, giving the overarching view. (P1)*  *I: OK, so you think that there should be doctors and scientists in the video? Would that be helpful?*  *P20: I I do think so yeah. And tell them that. Hey we have been working for many years in this industry as a doctor or a scientist and I can confirm this is good…I think having people who know their stuff would be much more…OK, Dr says it. Yeah, I believe in that. 'cause I trust Drs.*  *they will believe their pastors. (P17)*  *Try to get any religious leader because people trust them more. The Elder people trust them more so if you get the religious leader from each community. Oh, they're trusted. They're saying this, so you should follow them because for the older generation, they're more. They trust the religious leader more. (P4)*  *just putting it out there in different languages at different times…And putting it places that are accessible like you know, like the Asian channels and things like that… places like mosques and things. (P6)*  *Maybe going to places like the mosque, going to places like the church…if it can be done in other languages….(P17)*  *if you’re gonna use famous people then why not famous woman you know? Rather than two men…I would much prefer to have a younger person in the video, male or female (P17)*  *there were some kind of young adults, but the majority were what we call them aunties and uncles. (P16)*  *Different age groups…Maybe some students who can share their experience. (P15)*  *I think there needs to be more personalities on there from different genres. Like for the younger people. Someone from the music industry. I don't know someone like Dave or Stormzy or someone like that just to tap into that young age range. (P5)* | *One approach that might go some way to allaying the fears of BME communities would of course be to ensure that publicity materials aimed at encouraging BME people to take up the vaccine are fronted by BME medical practitioners. If BME nurses and doctors are seen to be accepting the vaccine at an overwhelmingly high rate this would, I'm sure, encourage greater numbers of people in the wider BME communities to do likewise.* |
| Who would benefit from seeing the film | *The elder people is gonna get more benefit from this video, yeah…because as we are young we can see like anyone in the film [and] we understand. But when the older people see our communities, our people saying the same thing, they're gonna understand more. (P4)*  *I think like there's some younger ones who's still haven't [been vaccinated]. Yeah, I think if they gave themselves a chance to watch the film, maybe just aim for the younger ones. The 20s and the 30s. The older folks are, you know, we know that when we get to a certain age, we know what is ahead of us and we want to protect ourselves as much as we can. Yeah, but the younger ones, you know. I think they need something geared at them so that it says well look you ain't gonna get to be the older age if you don't do what you're supposed to do to protect yourself. (P18)*  *For us, it doesn't matter, for our generation, you know. You look around white, black, pink, Chinese. Do you see what I mean? Because the information is there for us to read up about it. Look at it and understand it in whichever way. Whichever communities are involved. (P6)*  *People who know that there is COVID but don't know what exactly to do. Or what simple things need to be done, like for example some Asian housewives, they probably would not have access to services outside. Like a lot of Muslims I know have been truly homebound and they have limited knowledge. (P13)*  *I think for people who are neutral it will work. Definitely work. The people who are not neutral…so somehow they shifted to the negative side. Then we need to be little bit more. Present facts...say Don't believe those things. But I think for neutral people it will work definitely. (P20)*  *P10: It's like the people who would benefit are the ones who are Perhaps not clear on what to do.*  *Int: Yeah, won't watch it.*  *P10: Well, yeah, exactly. There's a spectrum of people and we've either got those at one end of the spectrum, who are curious, Interested… more engaged. Then you've got the other end where they've made up their mind. And those are really the ones you want to watch it.*  *'You’re talking to the wrong cohort of person. I mean gosh, we can get into…a whole different conversation, but people who are like me it wouldn't be an issue if there were black people and brown people on that video or not.’ (P19)*  *‘It's hard for me to say 'cause I'm not one of those people, so I mean, I'm a person of colour, but I don't I make my own. I've done my own thinking and I'm not influenced by a community.’ (P18)*  *‘I can't really comment on how convincing that would be because I can't put myself in their shoes.’* |  |
| Impact on behaviour | *The film doesn't speak to me. The film doesn't give me any fresh or new information to change my perspective, and that's really what I would have liked to have seen. (P1)*  *[It’s] not a bad thing. You know, sometimes reminding you ‘hey, you keep on doing what you’re doing’. (P20)* |  |
| Confidence in the vaccination programme | *In terms of the vaccine, I think there's got to be a lot more of a discussion forum and allowing both sides of the argument before many of us will be convinced to take the jab because right now we're only hearing one side of the discussion and the other side is being muted. And because of that it makes us feel something is not quite right here…We need to hear from those medical professionals who advocate for the vaccine and those medical professionals who don't advocate for it and we need to hear that discussion and be able to participate in that discussion for us, well, most people from African Caribbean communities to make a decision to take the vaccine, because it's about having informed consent and currently most of us do not believe that we are being given informed consent.*  *They’re not allowing that discussion, they're not allowing debate. They're not allowing people to, you know, the voice of dissent. They're not allowing that to be heard, so there must be a hidden agenda. So this is what feeds into this conspiracy theory now around COVID around the vaccines around the efficacy of the vaccines around the morbidity rate of the vaccines, you know. (P1)*  *I think they are pushing it too hard. Nobody wants to die. Why can't they let people you know decide on their own? (P2)*  *It scaremongers people into taking a vaccination…It doesn't explicitly tell people that... the vaccination doesn't stop you from spreading. It doesn't stop you from catching it. It needs to be balanced. Did you see what I mean? (P19)*  *Some of my colleagues. They still haven't taken vaccination and that surprises me, and I'm like why? they're like, well, there's not enough scientific study, or wait for everyone else to take a vaccine. (P20)* | *The whole threat/ coercion behind trying to mandate this vaccine adds to my suspicion as to its motive. Free will should be just that, free. A democracy that takes away this basic right has fallen into the dark ages.*  *Deep inside me, l believe government has a secret agenda.*  *Historically we have been guinea pigs for trial medicines…The fact that there is a rush, pressure, biased media and threats to take the vaccine raises great suspicion.*  *[The] Government is not to be trusted*  *I believe that black and ethnic communities have some justification for their statistically higher level of mistrust as to the purposes and efficacy of Covid vaccines. Given the somewhat shambolic UK central government's response to the Covid 19 pandemic together with historical instances of misuse of western medicines by colonial authorities that mistrust, in my opinion, is not without some justification.* |

**Table 4: Initial qualitive themes (coding tree)**

| **Category** | **Relevant themes** |
| --- | --- |
| **Practicalities** | - Background of participants - Motivation to participate in the survey - Accessing the survey and film - Watching and sharing the film - Guidance sheet |
| **Perceptions of the film** | - Understanding of key messages - Conveyance of messages - People in the film - Thoughts on the ethnicity-specific nature of the film - What participants liked about the film - Negative impressions/offensive aspects - Impact on behaviour - Who would benefit from watching the film? - Suggestions for improvement |
| **Covid in general** | - The value of perspective-historical and global - Protective behaviours - Social responsibility - Misinformation - Children - Confidence in the vaccination programme - Issues of trust - Changes over time |

**Table 5: Opinions of the film (questionnaire responses)**

| How likely are you to recommend the film to friends and family? | n | % |
| --- | --- | --- |
| Very likely | 37 | 28.5 |
| Fairly likely | 26 | 20.0 |
| Neither likely nor unlikely | 19 | 14.6 |
| Fairly unlikely | 11 | 8.5 |
| Very unlikely | 10 | 7.7 |
| No response | 27 | 20.8 |
| Total | **130** | **100.0** |
| How well do you remember what the film was about? |  |  |
| Very well | 44 | 33.9 |
| Quite well | 48 | 37.0 |
| Not much | 10 | 7.7 |
| Not at all | 0 | 0.0 |
| No response | 28 | 21.5 |
| Total | 130 | 100.0 |
| What is your overall opinion of the film? |  |  |
| Very good | 40 | 30.8 |
| Fairly good | 39 | 30.0 |
| Neither good nor poor | 15 | 11.5 |
| Fairly poor | 7 | 5.4 |
| Very poor | 2 | 1.5 |
| No response | 27 | 20.8 |
| Total | **130** | **100.0** |

**Participant Interviews Topic Guide**

**Introduction**-recording, consent, withdrawal, stopping interview

**Interventions**

1. Who provided you with a link to the film?
2. What did they say about it?
3. What could we do to improve the process of providing people with a link to the film so that they would be encouraged to view it?
4. What made you decide to watch the film?
5. Did you watch it alone or with other people?

**What is your overall impression of the film?**

Probes:

What did you like about it?

Was there anything that could be improved?

Would you be likely to share the film with people you know?

**From your perspective what were the key messages of the film?**

Probes:

What are the main points that you would take away from the film?

What are your views about the way the messages were communicated?

**Were there any elements that were difficult to understand?**

Probes:

Which were they? And why?

How could they be improved?

**Were there any elements that were offensive?**

Probes:

Which were they? And why?

How could they be improved?

**On the basis of watching the film have you changed how you intend to act to protect yourself against Covid-19?**

Probes:

What would you do now? And why?

Would you be likely to share the film with people you know?

Why do you think they would benefit from seeing the film?

**UNVACCINATED ONLY: On the questionnaire you completed you said that as a result of watching the film you had not changed you mind about being vaccinated against Covid-19**

1. **Is this still the case or have you since been vaccinated?**
2. **If you have been vaccinated what made you change your mind?**
3. **If you haven’t been vaccinated what are your reasons for this decision?**

If you know people who have not yet had their vaccination, would you be happy to tell them about the film and/or share the film with them?

1. If so, please tell me why?
2. If not, please tell me why not?

On the questionnaire you completed you said that you had been vaccinated. Are you planning to have a second vaccination and/or booster dose?

1. If so, please tell me why?
2. If not, please tell me why not?

If you have children between the ages of 12-15 have they been vaccinated?

1. If so, please tell me why?
2. If not, please tell me why not?
3. Will they be having a second vaccination?

Covid 19 guidance sheet did you get a link to it? If so did you look at it and what did you think? Was it useful? Was there anything you did/didn't like about the guidance sheet?

**Survey**

Who provided you with a link to the survey?

What did they say about it?

What made you decide to complete the survey?

What could we do to improve the process of providing people with a link to the survey so that they would be encouraged to complete it?

**Closing**-Anything else you would like to say? Reiterate withdrawal options, sending of voucher

**Participant Interviews Topic Guide (Version for vaccinated participants)**

**Full Title:** Developing and delivering targeted SARS-CoV-2 (COVID-19) health interventions to Black, Asian and Minority Ethnic (BAME) communities living in the UK. The COBHAM Study

**Short title**: Targeting health Interventions to BAME communities

**Principal Investigator:** Professor Aftab Ala PhD FRCP

Interventions

1. Who provided you with a link to the film?
2. What did they say about it?
3. What made you decide to watch the film?
4. Did you watch it alone or with other people?
5. What was your overall impression of the film?
6. What was the key message(s) you took away from the film?

1. If you know people who have not yet had their vaccination, would you be happy to tell them about the film and/or share the film with them?
2. If so, please tell me why?
3. If not, please tell me why not?

1. On the questionnaire you completed you said that you had been vaccinated. Are you planning to have a second vaccination and/or booster dose?
2. If so, please tell me why?
3. If not, please tell me why not?
5. If you have children between the ages of 12-15 have they been vaccinated?
6. If so, please tell me why?
7. If not, please tell me why not?
8. Will they be having a second vaccination?

1. If you have children between the age of 5-12 and the government advises vaccination for this group, how would you feel about getting them vaccinated?

1. What could we do to improve the process of providing people with a link to the film so that they would be encouraged to view it?

Evaluation process

1. Who provided you with a link to the survey?
2. What did they say about it?
3. What made you decide to complete the survey?
4. What could we do to improve the process of providing people with a link to the survey so that they would be encouraged to complete it?
